# "This does my head in". Ethnographic study of self-management by people with diabetes

**DOI:** 10.1186/1472-6963-12-83

**Published:** 2012-03-29

**Authors:** Susan Hinder, Trisha Greenhalgh

**Affiliations:** 1 Centre for Primary Care and Public Health, Barts and The London School of Medicine and Dentistry, London E1 2AB, UK

**Keywords:** Diabetes, Self-management, Structuration theory, Ethnography

## Abstract

**Background:**

Self-management is rarely studied 'in the wild'. We sought to produce a richer understanding of how people live with diabetes and why self-management is challenging for some.

**Method:**

Ethnographic study supplemented with background documents on social context. We studied a socio-economically and ethnically diverse UK population. We sampled 30 people with diabetes (15 type 1, 15 type 2) by snowballing from patient groups, community contacts and NHS clinics. Participants (aged 5-88, from a range of ethnic and socio-economic groups) were shadowed at home and in the community for 2-4 periods of several hours (total 88 visits, 230 hours); interviewed (sometimes with a family member or carer) about their self-management efforts and support needs; and taken out for a meal. Detailed field notes were made and annotated. Data analysis was informed by structuration theory, which assumes that individuals' actions and choices depend on their dispositions and capabilities, which in turn are shaped and constrained (though not entirely determined) by wider social structures.

**Results:**

Self-management comprised both practical and cognitive tasks (e.g. self-monitoring, menu planning, medication adjustment) and socio-emotional ones (e.g. coping with illness, managing relatives' input, negotiating access to services or resources). Self-management was hard work, and was enabled or constrained by economic, material and socio-cultural conditions within the family, workplace and community. Some people managed their diabetes skilfully and flexibly, drawing on personal capabilities, family and social networks and the healthcare system. For others, capacity to self-manage (including overcoming economic and socio-cultural constraints) was limited by co-morbidity, cognitive ability, psychological factors (e.g. under-confidence, denial) and social capital. The consequences of self-management efforts strongly influenced people's capacity and motivation to continue them.

**Conclusion:**

Self-management of diabetes is physically, intellectually, emotionally and socially demanding. Non-engagement with self-management may make sense in the context of low personal resources (e.g. health literacy, resilience) and overwhelming personal, family and social circumstances. Success of self-management as a policy solution will be affected by interacting influences at three levels: [a] at micro level by individuals' dispositions and capabilities; [b] at meso level by roles, relationships and material conditions within the family and in the workplace, school and healthcare organisation; and [c] at macro level by prevailing economic conditions, cultural norms and expectations, and the underpinning logic of the healthcare system. We propose that the research agenda on living with diabetes be extended and the political economy of self-management systematically studied.

## Background

Prevalence of diabetes is rising rapidly in most countries. Epidemiologists predict that within a generation, demand will outstrip supply for essential medical services [1]. Based on economic evaluations of randomised controlled trials, some researchers have estimated that educating people to self-manage their diabetes will significantly increase capacity in the healthcare system [2,3]. Neither self-management nor self-care has a universally agreed definition. The former is sometimes viewed as a complex construct which, in addition to the biomedical tasks of self care (defined as the various behaviours linked to managing illness and promoting and restoring health [4]), includes personal, cultural, social and political dimensions such as making sense of illness, rebuilding what sociologists have called "spoiled identity" after the diagnosis of a potentially stigmatising condition and lobbying for disability rights [5-10]. Lorig and Holman define self management as the full range of tasks undertaken by a person with chronic illness, including medical management, role management and emotional management [11].

The centrepiece of self-management policy in UK has been the Expert Patient Programme, which aims to increase independence and reduce use of health services [12]. Whilst self-efficacy has improved in people completing the Expert Patient Programme, improvements in disease outcomes have been modest or absent, especially in lower socio-economic and minority ethnic groups [13]. The programme had zero impact, for example, on type 2 diabetes in Hackney's multi-ethnic, inner-city population (Griffiths C, personal communication, unpublished results of MEDEA trial). Diabetes-specific structured education programmes include DAFNE ('dose adjustment for normal eating') and DESMOND ('diabetes education and self management for ongoing and newly diagnosed') for type 1 and type 2 diabetes respectively, which have led to significant improvements in disease outcomes compared to usual-care controls in controlled trials [14,15]. But they have been criticised for focusing too heavily on a relatively narrow biomedical skill set, and the transferability of such programmes to people with low health literacy and/or complex personal or social circumstances has been questioned [5,16,17].

Questionnaire studies have shown a positive association between psychological constructs (such as self-efficacy and locus of control) and effective diabetes self-management [6,18,19]. Researchers who invited people with diabetes to describe their experiences have concluded that self-management is culturally embedded, dependent on core knowledge and understanding, and improves with family and social support (see Discussion) [20-28]. Given the high research and policy interest in self-management, it is surprising that there are almost no recent research studies of self-management in individuals going about their daily lives. A classic sociological study of "living at home with chronic illness" was published in 1988, based on interviews rather than direct observation [29]. More recently, Korean researchers observed eating practices in 15 people with diabetes and concluded that cultural meanings were important in food choices [30]. An ethnographic study in Thailand of 33 people with diabetes found no evidence of "spoiled identity", perhaps because diabetes did not have visible manifestations in most people; but participants spent much time and effort in self-management work (diet control, exercise, clinic attendance, self-monitoring) in order to achieve what they described as "normality" [31].

People with diabetes spend around 1% of their time in contact with health professionals; the other 99% is virtually a closed book to clinicians and researchers. Our study sought to fill this evidence gap by directly observing how people live with diabetes in the home, family and community - and especially to identify the positive and negative influences on effective self-management.

## Methods

### Theoretical and methodological approach

**Figure 1:**
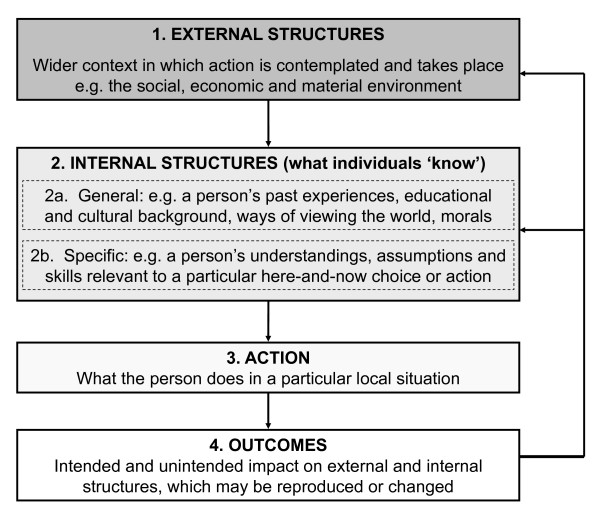
**Stones' version of structuration theory, showing four inter-related levels of analysis (adapted from **[33]**)**.

We adopted a sociological perspective drawn from structuration theory. In its original formulation, Giddens proposed a two-part (structure-agency) relationship: social structures (such things as social norms, moral codes, meaning-systems and political-economic institutions) both shape and constrain individual agency (people's actions and choices), which in turn serve to reproduce and shape social structures [32]. More recently, Stones has refined and extended structuration theory to accommodate the complexity and inherent contradictions of the social order in today's globalised, multicultural, networked and technology-dominated society [33]. He invites researchers to consider four aspects of the structure-agency relationship as it plays out in social situations: (a) external structures (the physical, social or economic environment in which actions are contemplated and take place, which are strongly influenced by an individual's position in society and which may be either enabling or constraining); (b) internal structures (what individuals 'know', including general life experience, educational and cultural background, and personal morals; and also the beliefs, assumptions, skills and capabilities that are relevant to a specific, here-and-now action or choice); (c) active agency (in which individuals draw on their stock of knowledge and experience and their assessment of a social situation to inform particular actions or choices); and (d) outcomes (which may feed back on both external and internal structures, reproducing and perhaps changing these). These are shown diagrammatically in Figure 1.

In this model, 'what individuals know' is not limited to their cognitive awareness of facts; it also includes perspectival or partial understandings (such as lay epidemiology), unconscious emotional urges (such as the desire to please an authority figure), and past experiences (such as a previous confrontation over 'bad' test results). In any social experience, the individual draws not merely on his or her own internal structures (what he or she 'knows' about the social world), but on their knowledge of the internal structures of other agents (i.e. what A thinks B 'knows'). For example, a patient from a particular socio-cultural background makes assumptions about what their clinician would expect, and tailors their own action accordingly.

Stones' adaptation of structuration theory thus combines an etic perspective (considering *external *social structures independently of individuals' understanding and perception) and an emic one (considering *internal *social structures via a hermeneutic study of what individuals 'know'), along with actions and outcomes. It is well suited to studying how people's actions and choices are situated within a particular meso- (home, family, organisation) and macro- (socio-cultural, political-economic) context. The study design recommended by Stones is ethnographic observation, supplemented by analysis of background data on the wider social context. The ethnographer immerses him or herself in a social situation and collects naturalistic data (i.e. real-world observations and naturally-occurring talk rather than 'under experimental conditions') in a pragmatic, reflexive and emergent way [34]. Ethnographic data are rich in qualitative description, allowing the researcher to interpret what is "really going on". Significantly, ethnography allows the researcher to capture data on what people actually do, rather than what they say they do. In this study, we sought to use an ethnographic approach to explain why individuals in different situations and settings took particular actions and choices relating to diabetes (including choosing *not *to undertake what a health professional would call 'self-management').

### Sampling and recruitment

In order to capture as wide a range of illness experience as possible, we sought a sample of 30 people with diabetes representing maximum variety by age, gender, ethnicity, health literacy, IT literacy, stage and severity of diabetes, level of family support and socio-economic status. We recruited from a range of settings including a local diabetes service spanning primary and secondary care, patient groups, community contacts and ethnic organisations in four counties in northern England. We used snowball sampling from our initial participants (i.e. we asked them to nominate another person with diabetes known to them). All participants gave written informed consent or assent. For the three participants under 13 and one adult with a mild learning disability, we used a specially designed assent procedure and form, and included the parents or designated carer in the consent process and the ethnography. Two participants were limited English speakers; they chose to have a lay (family member) interpreter rather than a professional interpreter.

### Data collection

Data collection occurred between 2008 and 2011. Our dataset was designed to inform the four analytic levels shown in Figure 1. In particular, we sought to inform both an etic analysis (from the researchers' perspective) of the wider economic, social and material context in which self-management took place or was contemplated, as well as an emic analysis (from the participants' perspective) of what individuals 'knew', including their cultural and educational background and the overall interpretive schema through which they made sense of their world, as well as the particular knowledge, abilities, practical skills, assumptions and desires which were brought to bear (or not) on the management of their diabetes. The data sources for the different levels of analysis are shown in Table 1.

**Table 1:** Data sources and analytic methods for structuration analysis of diabetes self-management

Level of analysis	Data sources	Analytic approach	Output
External structures	National policy and policy-related documents e.g. public health strategy, NICE guidance, agreements with the food industry Publicly accessible data on local demographics, disease patterns (from local public health reports) and built environment (food outlets, leisure centres) Index of multiple deprivation for locality Field notes on neighbourhoods, homes, workplaces, schools, food outlets	Thematic analysis of documents in historical context and texts. Findings drawn together by narrative synthesis to identify overall themes and key changes over time.	'Etic' understanding (from the researchers' perspective) of the wider structural context in which 'self-management' is contemplated and takes place

Internal structures	Ethnographic observations and naturalistic interviews with participant, including: Participant's explanations of what they were doing and why Participant's drawing of 'my diabetes' Participant's spontaneously disclosed beliefs, values and assumptions	Phenomenological analysis. Where a series of interviews was obtained from one agent, these were analysed longitudinally for change over time	Hermeneutic understanding of dispositions, perceptions and understandings of the index case and other relevant actors
			
	Ethnographic observations and naturalistic interviews with other relevant actors e.g. Parent and teacher (if a child) Partner or carer Friends Adult children		

Actions	Ethnographic observation of participant in activities of daily living at home, work, school Meal in a café Taking exercise (if this occurred) Visiting a health professional (if this occurred)	Interpretive analysis of actions in context, drawing on theories of symbolic interactionism and ethnomethodology	Understanding of why the agent-in-focus acted in particular ways in particular situations

Outcomes	Study of immediate consequences of action e.g. via direct ethnographic observation	Interpretive analysis of actions in context	Understanding of the short-term intended and unintended impact of social action

Many potential data sources were relevant to our etic study of context, but for practical reasons we limited the dataset to sources we considered to have the most direct bearing on the social, cultural and political context of self-management decisions and actions: [a] policy and policy-related documents on economic conditions, diabetes management, food labelling and promotion of positive behaviour choices; [b] our ethnographic observations in the home, neighbourhood and, where accessible, the participant's work or school environment (see details below); and [c] the Index of Multiple Deprivation (IMD) of the participant's home postcode. IMD is a composite score for a small neighbourhood, derived from aggregated responses to the ten-yearly national census and with some but not all components updated annually using local statistics. It consists of 38 indicators of deprivation grouped in 7 domains: household income, employment, health status and disability, crime, skills and training (including but not limited to formal education), barriers to housing and services, and living environment (including access to open spaces and leisure facilities). IMD scores for localities across the UK range from 0.4 to 84 (median 17.1); higher scores indicate more severe deprivation.

For the emic component of the study, we used three data sources, described in detail below: [a] ethnographic observation of the participant; [b] naturalistic interviews; and [c] a drawing of "my diabetes". We undertook between two and four periods of detailed ethnographic observation on each participant (230 person-hours of observation in all). We accompanied them for periods of 2-5 hours as they went about their daily life, noting issues to do with their condition as these arose in conversation, actions or events. We were interested in how participants managed their diabetes; what their information, communication and support needs were in relation to it and how they addressed them (or why they didn't). We noted any individuals (e.g. partner, other relatives, friends, peers, GP, specialist nurse) who helped the participant manage their condition and the technologies (e.g. blood glucose meter, insulin pump, telephone) which they or their carers made use of.

In half the cases, we observed the participant making a meal or snack (or one being made for them) and eating it in the home environment. We also took all but four participants to a local café or restaurant (selected by them) where they were invited to order a meal and a drink. This provided an opportunity to observe how diabetes affected their food choices and how (if relevant) they coped with measuring blood sugar and injecting insulin. Four participants invited the researcher to accompany them on visits to a health professional in relation to their diabetes (nurse, podiatrist, optometrist, pharmacist); in these visits, verbal informed consent was sought, and in all cases obtained, from the health professional for the researcher to be present. We went on walks with two participants and to the gym with one.

During the ethnography, we conducted naturalistic interviews (i.e. we interviewed the participant and any relatives or friends they chose to have with them, as they went about daily activities, and asked them to explain their perspective on things which came up spontaneously - for example why they chose a particular meal in a café, or why they were upset in a situation). Whist we did not use a formal topic list, we were particularly interested in knowledge needs for managing diabetes and information sources used to meet those needs. Rather, we used a conversational format, taking cues from things that interested or surprised us. To help engage the participant in the research, and to add to the diversity of data sources, we invited them to draw a picture of "my diabetes", showing themselves in the centre plus the people and technologies involved in managing the illness. We used the drawing activity mainly to engage participants (because people talked about their diabetes while considering what to draw), and some people preferred to describe rather than draw their illness. We made brief contemporaneous notes and spent several hours immediately afterwards annotating these and adding further recollections and reflections. This approach had the advantage of being minimally intrusive to participants but did not allow us to capture verbatim talk.

### Data interpretation and analysis

The different analytic approaches used for our large and diverse dataset are summarised in Table 1. Following Atkinson and Hammersley, who emphasise the value of observing naturally occurring talk and action and interpreting these in context [35], we placed greater significance on what people did, and how we observed them to react to events in real-life situations, than on what they said they believed, "would do" or "had done". To analyse our ethnographic notes, we used a traditional approach of immersion in the text through repeated reading (and studying any drawings in a similar way), thereby developing provisional analytic categories and iteratively refining these categories by the constant comparative method (comparing our analysis to date with new data as these emerged) [36]. To help achieve this, we used an Excel spreadsheet to aid initial data management and support a preliminary framework analysis to gain familiarity with the data, and Atlas Ti qualitative software for a more detailed thematic analysis, again chiefly to gain familiarity with the data before going on to apply the strong structuration theory approach shown in Table 1. Each researcher prepared an initial interpretation of several cases before meeting to combine these and resolve discrepancies through discussion; and this process was repeated as more cases were added.

To achieve the four-level analytic approach shown in Table 1, we first produced a description of the macro context, then considered the individual backgrounds and world views represented in our sample. Next, we considered particular actions or incidents relating to self-management in detail to explore how the possibilities available to the person were influenced by the macro economic, social, cultural and material context, how this context shaped and constrained their actions and choices in this incident, and how the outcomes of action (or inaction) fed back into the system at both micro and macro level. This involved both individual reflection by both researchers and discussion between us. We offered participants the opportunity to hear our interpretation of their case verbally, or see it in writing, and feed back comments or factual corrections We anonymised cases for publication using ethnically appropriate pseudonyms and an adaptation of the critical fiction technique (i.e. we systematically fictionalised identifying details in a way which retained essential themes of the clinical case) [37].

## Results and discussion

### Description of study participants

Our 30 participants ranged in age from 5 to 88 (median 48.5); 22 were white British, 7 British South Asian (two of whom were limited English speakers) and one African. 22 were taking insulin, of whom 15 described themselves as 'type 1'. Duration of diabetes ranged from 1 to 48 years (median 9). Only 7 of the 30, including the five aged 16 or under, felt they had no other illnesses apart from diabetes; 17 described complications of diabetes. The index of multiple deprivation (IMD) score of their home postcode ranged from 8.6 to 76.1 (median 40.6). Of the 25 participants over 16, 8 were in paid work, 4 retired and 11 drawing benefits; three preferred not to say. Three were educated to degree level and four more had A-levels; the others had secondary education and/or diploma certificates or preferred not to say. In sum, this sample broadly reflected the demographics of people with diabetes, including the higher prevalence of this condition in Asians and in lower socio-economic and less educated groups. It was deliberately skewed towards people treated with insulin (since self-management raises additional challenges in this group).

### The wider context for self-management

Our etic analysis revealed a number of overlapping external social structures (political/policy, economic, socio-cultural, health system) within which the opportunities for, and constraints on, self-management were nested. We briefly outline those relevant to this study of self-management. Our field work occurred at a time (2008-11) of negative economic growth, including the worst recession since the second world war in 2009-10 (Office of National Statistics data). Unemployment levels rose over this period, as did job uncertainty, food and fuel prices, public transport costs, and personal debt - and these disproportionately affected the poorest tenth of society [38]. However, this was also a period of relatively generous funding for the National Health Service, when general practitioners were being offered strong financial incentives to prevent and manage diabetes; significant improvement in diabetes outcomes nationally occurred in this period [39].

UK health policy throughout our data collection period emphasised individual responsibility for lifestyle choices rather than social determinants of health. For example, whilst the 2010 white paper *Healthy Lives, Healthy People *promised a "radical new approach" to public health based on life course epidemiology and talked in somewhat vague terms about "empowering local communities" [40], actual policies were based largely on behavioural economics and emphasised strategies to "nudge" individual behaviour through incentives, prompts and health education [41]. In the Public Health Responsibility Deal, for example, the food industry was encouraged to provide healthier eating options on a voluntary basis [40].

### Individual dispositions and capacities for self-management

Our ethnographic observations illustrated how the wider social, economic and cultural context described above influenced self-management via individuals' social position and via the cultural and material resources available to them. Importantly, however, these external structures did not wholly determine self-management behaviour, since our 30 participants varied considerably in the type of people they were and in the personal resources (abilities, skills and traits) they were able to bring to bear on self-management in any given context. For example, they differed widely in education, personality, ambition, motivation, cultural perceptions and beliefs, religiosity, self-confidence, health and information literacy, and how they perceived and framed their condition. Seven (including one with a diagnosed mild learning disability) described themselves as "slow" or having "memory" or "understanding" difficulties. Five had evident and severe disabilities such as paralysis, blindness, or heart failure, which made them dependent on others for basic care needs. Four more were largely housebound through a combination of non-specific disability, obesity, depression or low mood, generalised tiredness, sleep apnoea and lack of fitness. All these differences affected people's capacity and motivation to self-manage and how they went about this.

### The work of self-management

List: What people did when they 'self-managed' their diabetes

PRACTICAL AND COGNITIVE TASKS

Monitoring bodily symptoms (indicative of high or low blood glucose, high blood pressure, "stress") and taking steps to feel normal again

Shopping

Preparing meals (sometimes separately from the rest of the family)

Exercising (usually, walking or attending gym)

Injecting insulin or taking tablets

Calculating variable insulin dosage by estimating nutritional content of food

Measuring and recording blood glucose levels

Coping with 'hypos' or preventing them (by ensuring that they took food and drink when going out)

Maintaining equipment e.g. replacing needles on insulin pen

Seeking general information on diabetes from the Internet or libraries

Seeking specific information relating to own diabetes e.g. from health professionals

SOCIO-EMOTIONAL TASKS

Coping with feelings about diabetes (including anger and frustration)

Making sense of information or advice from health professionals and/or seeking confirmation of this from peers or other professionals

Maintaining smoking cessation e.g. avoiding situations where they might be tempted to smoke

Explaining to relatives, friends and work colleagues what diabetes is and how it is managed

Training relatives or work colleagues how to manage diabetic emergencies

Seeking and/or offering advice and support in diabetes peer support groups (e.g. Facebook)

Negotiating access to health care (e.g. seeking appointments or referrals)

Obtaining resources (medication, blood testing strips, blood glucose meter)

Negotiating the diagnosis, symptoms or management of diabetes with health professionals (including persuading doctors and nurses to engage with their self-management efforts)

Parents persuading children (and family members persuading the adult with diabetes) to consume recommended food/drink and discouraging them from consuming 'bad' foods

Parents managing wider family dynamics (e.g. siblings feeling cheated when the family diet is restricted to align with the diabetic child)

A strong over-arching theme in our data was the perception that self-management involved physical, cognitive or emotional *work*. By 'work' we mean an ongoing need to put in effort in order to achieve physical wellbeing and a sense of autonomy and social worth. A few expressed this directly, using metaphors like "hassle", "grief" or "ball ache" (see quote below). The list above shows examples of self-management tasks we observed directly, which correspond broadly to Corbin and Strauss's categories of 'illness work', 'identity work' and 'everyday life work' [29]. Much time was spent on practical tasks such as blood glucose testing (which some found painful and stressful), planning and preparing meals, matching food intake and energy expenditure with medication, and caring for feet, as well as on socio-emotional tasks including negotiating access to health services; obtaining resources for their diabetes care; and managing (i.e. encouraging, resisting or moderating) the input of family, friends and colleagues.

A few insulin-treated participants drew confidently on an extensive knowledge base to make adjustments in their medication so as to accommodate variations in diet and activity.

'Tina orders a panini. [..] She says she'll get her plate of food and look at it and decide the size of dose [for her insulin pump]. She informs me that there are 2 different ways of giving a bolus - all at once, for something very sugary such as fruit juice, which will cause blood sugar to increase straight away. Something like a white bread panini needs a half an hour split dose - 30% of the dose straightaway and the remainder over the following half an hour. Depending on protein, fat or fibre, she needs to increase the length of time the bolus is given over.'

Field notes and interview from café visit with Tina, age 26, type 1 diabetes for 20 years

Tina had had little formal education and lived in a deprived locality (IMD score 32); she worked as a healthcare assistant. But she had recently engaged enthusiastically with an intensive diabetes education course and considered her life "transformed" by the pump. When asked for an image to depict her diabetes, she suggested "a picture of me with diabetes always there, but in the background". In short, she was competent in the work of self-management and this work was effective in achieving her goal of dietary freedom.

Insulin-treated participants with limited knowledge and/or confidence sometimes achieved 'self-management' by maintaining a strict and invariant dietary routine.

'He said his mum used to give him spinach and ricotta cannelloni (microwaveable) and half a loaf of bread to take for his lunch. But after a year he got fed up of spinach and ricotta cannelloni butties every day. He asked his mum to get him something else so she got him beef lasagne (microwaveable) for the next six months.'

Notes from naturalistic interview on visit to Callum, age 28, type 1 diabetes for 17 years

Whilst Callum had subsequently discovered greater dietary freedom following a DAFNE course, his metaphor for diabetes was still "a vice" (meaning, we assume, that he had to put in considerable effort to achieve this freedom). Between these extremes, many insulin-treated participants had developed a simplified dietary regimen driven by a few rules of thumb.

Many participants talked of "balance". But in contrast to the biomedical meaning of this term (physiological homeostasis), they saw self-management as relating to balance in their wider lives, including controlling personal stress levels, nurturing family and social relationships and achieving work-life balance. One aspect of "balance", for example, was between the immediate physical pleasure and social significance of food treats and the deferred benefits of a strict dietary regimen. Another was the balance between being open and transparent about the work of managing their diabetes and not burdening their friends and relatives with these issues.

### The 'meso' environment of the home, family and social group

Our 88 visits to participants' homes revealed striking differences in the material and social context for self-management. A few had comfortable houses which were well-equipped with working technologies, including broadband Internet and landline telephone, and located in settings close to fresh food sources and open spaces. But many of our participants enjoyed few or none of these benefits. There was a wide range of domestic arrangements from living alone (single, divorced or widowed); shared occupancy; nuclear, single-parent or 'blended' families; and multi-generational extended families. Participants' homes differed greatly in the availability of food - and in the symbolic meaning which food held within the family.

'Very large bowl of sweets on the bookcase. Large bottle of Coke plus large jar of toffees on a small table at the side of Wendy's armchair. Terry's Chocolate Orange on small table at the other side of the armchair plus a bottle of tomato sauce. [..] Wendy explains that her mother (aged 79) buys her loads of "goodies" e.g. recently brought back 3 carrier bags full of biscuits, sweets, cakes, joints of meat, burgers, etc. She has tackled her Mum before about giving her and her children so much sweet stuff but her response is "well, kids love goodies".'

Field notes from visit to Wendy, age 58, type 2 diabetes for 18 years, is obese and has had two strokes

For many participants, the prevailing material and social conditions within the home and the immediate neighbourhood provided challenges to effective self-management (Table 2). The impacts of poverty and family disruption were sometimes multiple and mutually reinforcing. Karl, for example, lived with his mother in a 2-bedroomed council house (IMD score 58); his brother lived with his father.

**Table 2:** Challenges to self-management in the home environment

Feature	Potential impact on self-management
Presence of sweets, sugared drinks and other health-negative foods, perhaps supplied by relatives as "gifts"	Difficulty following diabetes diet

Cramped housing	No dedicated place to keep diabetes monitoring equipment

Multiple occupants in the home, sometimes from several generations	Psychosocial stress from limited privacy and intergenerational conflict, with [perceived] impact on blood glucose control, blood pressure and lifestyle choices e.g. alcohol, smoking

Social problems within family (e.g. family member involved in drugs or crime; unemployment; domestic strife)	Psychosocial stress as above; family members less able to support the person with diabetes

Computer not working	More difficult to access health information and advice

Financial pressures	Food choices made primarily on the grounds of cost rather than nutritional value

Conflict with neighbours e.g. noise through walls	Psychosocial stress as above

Crime or fear of crime	Reluctance to exercise outside the home

'I ask if Karl goes to any activities outside school. He says he used to go to boxing but his dad lost his driving licence and they had to stop for three months and didn't start again.'

Field notes from visit to Karl, age 11, type 1 diabetes for 4 years, parents divorced

Whilst only a minority of participants were in paid work, many had domestic and/or carer duties, did voluntary work or were studying for a qualification. The various self-management tasks had to be fitted in with these wider commitments. Below, we describe a case example to illustrate the complexity of this:

Beryl, aged 50, has had type 2 diabetes for 15 years. She lives on a council estate (IMD score 58) and is on state benefits. She has hypertension, hypercholesterolaemia, retinopathy, neuropathy and had a gastric bypass for morbid obesity two years ago. Beryl left school with few qualifications and can barely use a computer. She signed up for a further education course two years ago but had to give this up to care for a sick relative. She recently resumed the course following the relative's death. Beryl's adult children have various social and financial problems (including fear of crime on another local estate) and depend heavily on her for childcare and financial and emotional support.

Beryl views diabetes as one of numerous interacting stressors in her life. During a home visit, Beryl takes a piece of paper and writes "Things that are doing my head in today". Prompted by the researcher, she writes a list of current issues in her life down the left hand side of the page and scores them where 1 = worst impact and 10 = no impact. "[adult daughter] - 3/10, [adult son] - 4/10; new puppy: 2/10; college course - 5/10; diabetes - 2/10; losing eyesight - 1/10; pains in feet - 1/10; shopping - 3/10; cleaning - 7/10; looking after grandkids - 1/10."

Before her gastric bypass operation, Beryl had too little energy to take exercise. She had been on escalating doses of insulin and her attempts to lose weight had failed; she had abandoned her diet and described herself at the time as a "chocoholic". At that time she did not even have the motivation to bath regularly or clean the house. Following bypass surgery, she was delighted to have lost a third of her body weight. She has begun to follow a much stricter diet, which involves cooking separately from her live-in relative (whom she says now has to "fend for himself"). She has also given up smoking and manages to fit regular exercise around her family and college commitments, even recently competing in a 3-mile charity walk ('Race for Life').

Partners played a very variable role in the management of participants' diabetes. Some took lead responsibility for the work of measuring and monitoring blood glucose, administering medication and supporting the individual in following their diet. At the other extreme, some partners appeared to demarcate diabetes management work, and the knowledge and skills needed to undertake it, as beyond the bounds of their relationship. Some partners viewed self-management of diabetes in moral terms (because neglect of one's own care had implications for the couple or family). In such circumstances, self-management was a potential source of conflict.

'Steve has not done his blood sugar today - he's just done "guesstimates". [...] Steve orders a pint of beer and (later) a pint of shandy. Steve and Lucia have an intense discussion about how she feels about him not managing his condition properly, because she sees it as an insult to her that he's not prepared to do all he can to be well and stay around for her. Lucia says people close to them have warned her off having a relationship with a person with diabetes.'

Field notes from home visit and restaurant outing with Steve (age 32, type 1 diabetes for 26 years, microvascular complications) and his partner Lucia

A key socio-emotional self-management task was managing partners' and other relatives' level of involvement with their diabetes, and in particular dealing with relatives who either refused to engage with the recommended restrictions on diet and lifestyle or sought to enforce them rigidly.

'He only gets Flora [margarine] on the toast if his wife makes it, he gives himself butter if he makes it. [...] Tonight his wife is away; he will have chunky soup with some sausage leftovers from last night sliced into it.'

Notes from naturalistic interview on visit to Vic, age 61, type 2 diabetes for 6 years, hypercholesterolaemia

Interestingly, in this example and that of Wendy (see above), the participant chose a 'workaround' (i.e. they accepted an implicit dietary arrangement when the relative was present but did not adhere to this in their absence) rather than continue to confront them about it. Others preferred to stand their ground and resist family pressure. Saleem, for example, was the patriarch of a traditional South Asian family; in this particular meso environment his relatives were less able to enforce their views on what he should eat.

'Saleem's son comments that he's on insulin because of his bad habits. I ask what the bad habits might be. Sweet, sugary things. Saleem says "they can't tell me I can't have them". [...] He says he has something sweet every day. His son says he's "zithy", the Urdu word for stubborn.'

Field notes from visit to Saleem, age 88, type 2 diabetes for 15 years

Some participants drew considerable practical or emotional support from friends, neighbours and extended family. Even when this support was limited, participants' self-management actions and choices were strongly influenced by their desire to act appropriately and keep face within their social group. This was particularly evident in teenagers and young adults.

'He tells me he plays football and goes to the gym. He doesn't make any special preparation for doing sports. Mum says he takes Lucozade with him. Asghar insists he doesn't and then Mum says he drank a whole bottle before football. She gets frustrated with him "What about the time I chased after you because you'd taken four bottles!" "I was taking them for my mates" Mum looks disgruntled - "They're too expensive to give them to your mates".'

Field notes from home visit to Asghar, age 16, type 1 diabetes for 7 years, IMD score 67.1

Lucozade is a commercial carbonated carbohydrate drink which many participants used to treat hypoglycaemic attacks, but which is also marketed as a sports drink. By handing out bottles to his friends, Asghar may have successfully de-medicalised his treatment and achieved social gain, but this trade-off had a very different social meaning for his mother, who was struggling to feed a family of six on state benefits.

A minority of participants were members of diabetes patient groups and/or online peer support networks where they exchanged experiences and tacit knowledge with other people with diabetes. Below, we reproduce examples of postings on a diabetes patient organisation public-access bulletin board used by one of our participants (identifying details have been fictionalised).

"Hi

the best way to get out of a low, is sugar and milk. trust me... and also quick question... can anyone tell when there blood levels are high, cus its obvious when there low but my doctor said i couldnt tell when they are high,, but i can: D: D... can anyone else?????"

-------------------------------------------------------------

"Hi All, i was wondering if some one could give me some advice about the pumps. i am 32 and a type one. i have tried everything under the sun to control my levels. i eat well, work out and take good care of myself AND still i cant get control myself. i feel rather alone and scared esp after reading some things online ref..."

"Hi i'm in the same position as you, i just cant get my sugars under control (its been an uphill struggle for 22 yrs). I have asked my consultant about a pump for several yrs but she always just ignored me... but finally last aug she said she would consider it !! In nov i was told that i have been accepted, but that there is a 2 yr waiting list. so i'm waiting. I've read that it is very hard work at first but the benifits are well worth it. My advise to you would be to first of all speak to your G.P/consultant/D.L.S [Diabetes Liaison Sister] and see what they say. If you meet all the NICE requirements (which it sounds like you do), keep on asking your consultant. Once you have got your consultants backing, your local PCT have to fund it! but in some areas they drag their feet because they dont want to part with the money. If this happens speak to your local MP (that's what I'm doing) and just keep on nagging. try looking at these websites: -http://www.nice.org.uk/TA151http://www.input.me.uk..."

"Try our online diary - record your glucose levels and the carbs you are eating online so they are all in one place http://diabeticfriend.co.uk/ I found it really useful to understand my levels while I was pregnant. Xxx"

-------------------------------------------------------------

"After my last check at hospital clinic they were concerned about my blood preesure as it was a tad on the high side and am already on medication for it. They asked my GP to keep an eye on it. First reading 155/70, 2 weeks later 152/70. I told receptionist that was too high as a person with diabetes should not have a reading that high, not above 130"

"Maybe if you brought a blood pressure machine. Take it daily and show your GP the results when you see them again. Personally I dont always rely on the receptionists. Good luck and hope you are settled soon."

---------------------------------------------------------------------------

"I just gave my 4 year old daughter 8 units of novorapid instead of 8 units of lantus can any1 let me know wot 2 do."

"Been there! If you have any milkshake or juice or junky food I'm sure she can manage something... x"

"I gave my son 28 units of novorapid by mistake 1 night, it was a very long nite, making sure he was ok, won't make that mistake again"

### Self-management outside the home

A recurring theme in our data was how much easier people found it to manage diabetes when they were in a familiar environment (usually but not always their own home) and following a regular routine. Settings outside the home were inherently more challenging and experienced as harder work. Some participants commented that their diabetes was more difficult to control at weekends because life was less predictable then. Several led restricted social lives and were reticent to go out to places with which they were unfamiliar.

We had hoped to follow people with diabetes in the work environment, but only one (a semi-retired administrator who worked part time for a small company) was prepared to be shadowed at work. The reason for this seemed to be that having a researcher accompanying them would have made the diabetes more visible in the workplace whereas the individual preferred not to draw attention to it. Several participants said they had given up a job because the "stress" had interfered with their diabetes control. Three issues seemed to contribute to this problem: intensity and unpredictability of workload, inability to control timing of meals, and shift work. Three participants described coping with a particular job until a 'squeeze' occurred (reduction in staffing or increase in expected performance). Two had given up nursing or nurse training after developing diabetes.

In contrast, the one school we visited appeared keen to accommodate and support the diabetic pupil. Staff were knowledgeable; a formal policy on the condition was evident; and the child's parents had made great efforts to engage staff in the strict routine needed for optimal diabetes control.

'At 10 am she gives William the biscuit his mum has brought in. Mum always wraps it in silver foil so it's very noticeable and puts it in the middle of the teacher's desk.'

Notes from naturalistic interview with class teacher of William, age 5, type 1 diabetes for 1 year

Many participants found eating out a strain because it was impossible to predict what the nutritional content of the meal would be or assess this accurately once the meal arrived. Several insulin-treated participants chose to eat out at corporate chain outlets such as McDonalds because these offered a detailed breakdown of nutritional content on their website (enabling the insulin dose for a particular menu choice to be worked out in advance). In the light of these findings, it is not surprising that several participants abandoned their otherwise tight dietary regimen when we took them out for a meal. Some but not all were reluctant to inject in cafés or restaurants either in the public areas or in the lavatory, since neither space was considered sufficiently "private". Injecting insulin was not a value-neutral medical procedure but a social practice which people with diabetes deemed appropriate or inappropriate in different contexts.

None of the food outlets we visited in this study offered a 'diabetic' option, and even when some nutritional information was given, it was inadequate to inform a titration of insulin dose. When questioned about a menu item, waiting staff were typically able to explain what a meal would look or taste like and whether it would contain nuts, but could give no indication of its total energy, fat or carbohydrate content.

Participants offered examples of limited understanding of diabetes by staff in other organisations. One person with an insulin pump, for example, described a humiliating episode at an airport where he had been strip-searched by security staff. Since then he has decided not to take his pump on holiday.

### The healthcare system and the self-managing patient

The main hospital diabetes service covering the localities in our sample was delivered by a multidisciplinary team and included free structured diabetes education and a telephone 'Care Call' support service.

'The Care Call system is that someone (non-clinical) phones her once a month. Four days before the call, people have to take blood sugars 4 times a day. The figures are then passed on to the diabetes nurse who will then phone to say whether to increase, decrease or stay the same with the insulin. Pauline can phone the service with any concerns. She says she phones them all the time.'

Notes from naturalistic interview on visit to Pauline, age 62, insulin-treated type 2 diabetes for 6 years

Pauline appeared particularly to value the responsive nature of the Care Call support service. For example, when she was booked for a hospital admission for an abdominal operation, the surgeon had dismissed with vague reassurance her questions about what would happen with her diabetes. The Care Call nurse had explained in detail how an insulin-treated patient would be managed on the surgical ward.

Almost all general practitioners in the locality received the maximum incentive payments for diabetes care. But whilst services appeared to be of a high standard overall, some participants experienced difficulties with individual practice staff whom they felt did not recognise or value their knowledge, showed little interest in their self-management efforts and sometimes seemed actively to discourage such efforts. One participant was in the process of changing his general practitioner because the practice nurse "did not like his questions". Such accounts were sometimes borne out by our own observations.

Optometrist: "Are you diabetic?"

Harry: "Yes".

Optometrist: "On tablets or insulin?"

Harry: "I'm on tablets and insulin."

Optometrist: "You're type 1 then."

Harry: "No, I'm type 2."

Optometrist: "Well you must have become type 1 now"

Field notes on accompanying Harry (age 73, insulin-treated type 2 diabetes for 2 years) to optometrist

Participants who sought actively to self-manage greatly valued continuity of care with a particular doctor or nurse. and saw them as pivotal in aiding the self-management. From their perspective, effective conversations about self-management could occur only when the health professional acknowledged their knowledge and expertise and trusted their account - and this was rarely the case in the absence of an established relationship.

### Observations of 'non self-management'

The ethnographic method allowed us to observe what we interpreted as 'non self-management' - that is, situations in which the self-management practices listed above might have been employed but were not. We were not surprised to observe some of our participants eating energy-dense, nutrient-poor ('junk') foods; snacking (e.g. on glucose tablets when not hypoglycaemic); not taking account of portion size; missing meals; not testing blood glucose levels even when they suspected them of being very high or low; managing (presumed) hypoglycaemic attacks by drinking large amounts of Lucozade from the bottle; smoking; consuming sugary foods and drinks to counteract the known hypoglycaemic effect of alcohol; and not recognising or addressing what we viewed as self-management needs which emerged in daily life. Many ordered high-sugar drinks when out for a meal even though 'diet' options were available. Few took regular physical exercise beyond household duties. Some omitted their prescribed medication (because of side effects) or insulin (to control their weight or reduce the risk of hypoglycaemic attacks when going out).

The underlying reasons for episodes of 'non self-management' were varied and complex. An important influence was the numerous other facets of people's daily lives (such as childcare, domestic duties or the restrictions and demands of paid employment); competing demands on a limited family budget; the constraints of co-morbidity; and the material and social contexts in which particular self-management tasks were (perceived as) more or less achievable and socially acceptable. Some participants found the requirement to plan menus, restrict dietary content, adjust medication dosage and keep written records of their progress difficult and dispiriting, especially in the context of psychosocial stress, complications (e.g. visual impairment, cognitive impairment) or co-morbidity.

'I ask if she counts carbohydrates. She says yes but says "it doesn't sit comfortably in my head". Melissa's husband tells me "it's a ball ache" to keep the log of blood sugars and food. He says it's doing his head in having to write down everything in the log. He's been doing it for the last 6 months.'

Field notes from home visit to Melissa, age 38, type 1 diabetes for 28 years, pregnant with multiple diabetes complications including severe visual impairment

In one participant, a pattern of insulin omission was linked to denial of diabetes in the context of high psychosocial stress and low health literacy. The individual had lost weight dramatically recently and insisted that she was not "dependent on" insulin any more. (With her consent, we supplied her with a letter of concern to take to her GP, and also informed the practice).

Some participants viewed self-management mainly or exclusively in terms of responding to bodily symptoms (a finding others have described previously [42]). Some considered routine diabetes check-ups "pointless" if there was no direct symptomatic improvement as a result. Participants who smoked generally justified this in terms of coping with "stress" and/or by denying the risks. Health-negative food choices were often described as "treats" and taken in what participants considered to be small quantities. In many cases, these were justified in terms of their social significance - for example at a social event or as part of a positive relationship with a family member or friend.

Some participants who had not attended self-management education told us that their current self-management regimen had been approved by a health professional. This affirmation was typically depicted in vague and non-specific terms.

'I ask was he given any information on diet. He says there was "a slight mention". Don't have greasy foods, don't have too many sweets. He saw the dietician who apparently told him that he wasn't doing anything wrong.'

Field notes on visit to Kamlesh, age 61, type 2 diabetes for 2 years, overweight with heart disease

### The consequences of self-management (and non self-management)

Structuration theory proposes that the outcomes of human action feed back to reinforce or change internal and external structures (Figure 1). One of the most powerful influences on participants' attitudes towards diabetes and their consequent self-management activity was the extent to which their efforts were effective.

The case example of Beryl (see above) illustrates how the consequences of self-management can feed back either positively or negatively to change internal structures. Before her gastric bypass operation, Beryl's attempts to lose weight had failed, creating a vicious circle of low motivation, poor eating, weight gain and increasing insulin dose. But with an altered metabolic profile, her self-management efforts had begun to bear fruit and the circle became positively reinforcing; she took increasing control of her diet, exercise and smoking despite adverse contextual influences.

A number of other participants had reversed a previous "chaotic" pattern by moving to an insulin pump or by going on a DAFNE or DESMOND course. As their efforts to control their diabetes became more successful, they described greater motivation and effort in self-management activities. In contrast, both Mary, aged 58, with poorly controlled insulin-treated type 2 diabetes, obesity and multiple complications (who described her diabetes as a "monster") and Priya, aged 14, with recurrent hypoglycaemic attacks with fits (whose parent described her diabetes as "a box of scary things") had made several unsuccessful attempts in the past to adjust their diet and medication.

### Summary of findings

This ethnographic study, in which a total of 88 extended visits were made to 30 people with diabetes in their homes and community, has shown that self-management comprised both practical and cognitive tasks (e.g. self-monitoring, menu planning, medication adjustment) and socio-emotional ones (e.g. coping with illness, managing relatives' input, negotiating access to healthcare or self-management resources, and making evaluative judgements about immediate versus deferred benefits). All this was hard work and time-consuming for people with diabetes and their families. Some managed their diabetes skilfully and flexibly, drawing on personal capabilities and family and social networks. For others, capacity to self-manage (including overcoming contextual barriers) was limited by co-morbidity, cognitive ability, psychological factors (e.g. under-confidence, denial) and social capital. As predicted by structuration theory, economic, material and socio-cultural conditions within the family, workplace and community sometimes created an enabling context for self-management and at other times provided significant barriers to achieving it.

'Non self-management' tended to occur in contexts where people's material, intellectual or emotional resources were stretched, including poverty, low health literacy, a demanding family or social context, or multiple co-morbidity - and especially when all these factors were present and interacting. Some healthcare staff appeared reluctant to accommodate advanced knowledge and/or self-managing attitudes and activities in their patients, especially when they did not have an ongoing care relationship with them. Food labelling in cafés and restaurants was inadequate to allow people with diabetes to apply advanced insulin dosage algorithms even when they were confident to use these.

### Strengths and limitations of this study

The main strength of this study is that it is, to our knowledge, the first detailed observational study of all aspects of self-management, including the context in which it occurs, in a sample of people with diabetes selected for maximum variety in both medical and socio-economic variables. The ethnographic design enabled us not merely to see self-management activities in family and social context, but also to study the multiple interacting influences on the under-researched phenomenon of *non *self-management.

Significant limitations of the study are that whilst we gained extensive access to people's homes, there were many aspects of personal and family life which we did not witness; some of our observations may have been influenced by the presence of the researcher; and we had limited access to the workplace. Furthermore, people may have behaved differently while being observed, though they might be expected to amend their behaviour towards what they considered to be compliance with professional expectations of self-management (e.g. to try to be seen to be following a diabetes diet). Whilst we still collected multiple examples of participants choosing not to self-manage, our findings should be interpreted in the light of this possible bias.

### Links to previous research

Our findings support and extend those from previous studies (most based on narrative interviews of people with diabetes, and a single ethnographic study) which highlighted the need to align the self-management agenda with the social demands of people's everyday lives; their need to maintain a coherent identity and a "normal" social life; and the finding that poverty and the physical and social environment may impact on self-management [21,25,31,43]. In particular, our findings resonate strongly with those of a previous study by our team which highlighted a number of 'storylines' within which the practical tasks of self-management acquire social meaning and moral worth, including rebuilding spoiled identity, living a disciplined and balanced life, mobilising a care network, navigating and negotiating in the health care system, and making ethical choices (such as allocating a limited family budget) [25]. They also align with work by an Australian team who identified eight dimensions for chronic disease self-management, including positive and active engagement in life, health-directed behaviour (e.g. dietary choices), self-management skills, health services navigation and social integration and support [44].

It is worth comparing our findings with those of qualitative interview studies linked to trials of self-management education. In the DAFNE trial, three qualitative interviews were conducted with each of 30 participants over a 12-month period [27,45]. These suggested that opportunities presented by more sophisticated self-management regimens for greater dietary freedom were counterbalanced by new challenges and burdens (e.g. having to simplify food choices). Researchers who interviewed 36 people recruited from a DESMOND course classified them into four 'types' depending on the degree of personal responsibility they took for their self-management activity [46].

It is perhaps unsurprising that the study reported here, which sought to study self-management in all its messy and idiosyncratic detail and take account of its multi-layered context has failed to explain participants' behaviour solely in terms of psychological states or traits. Our findings resonate more closely with the social ecology model offered by Glass and McAttee, in which psychology is just one of many dynamically interacting influences [47]. Whilst our work supports a conclusion that psychological constructs such as low motivation, low self-efficacy and external locus of control are significant mediators and moderators of diabetes self-management efforts, it also supports a more sophisticated, 'nested determinants' model in which these traits are in large part the product of prevailing social, economic and material circumstances.

## Conclusion

### Implications for policy and practice

Our findings suggest that the success of self-management as a policy solution will be affected by interacting influences at three levels. At the micro level, self-management depends crucially on individuals' dispositions and capabilities, and will have relatively little purchase in those with low health literacy and other relevant capabilities. At the meso level, self-management depends on key roles, relationships and material conditions within the family and also on the presence of a supportive infrastructure in the workplace, school and healthcare organisation. And at the macro level, self-management is likely to be influenced by prevailing economic conditions, cultural norms and expectations, and the underpinning logic of the healthcare system (e.g. the extent to which support for self-care is an expected and adequately remunerated aspect of the service). People's self-management efforts, in turn, will influence the meso environment of the family and (more indirectly) the organisational and wider social structures which support this practice (or not).

Many authors have argued that structured education for self-management should be supplemented with attention to the wider environment [5,13,17,48-50]. The Robert Wood Johnson Foundation in the USA, for example, has proposed a multi-level set of resources and support for self-management which include individual components (self-management education, continuity of clinical care, collaborative goal-setting, personalised support and troubleshooting) as well as community development (e.g. food outlets, safe exercise spaces), community health support workers, and health system development. Instruments to evaluate such multi-level resource packages have been developed and validated [51,52].

In contrast, and notwithstanding a small but politically high-profile demonstration project to encourage shops in low-income areas to sell more fresh fruit [53], self-management policy in the UK remains relatively narrowly focused on the person with chronic illness and assumes that the key focus for change is that individual's knowledge, attitudes and behaviour. The findings reported here suggest that a much broader approach should be taken. We need to develop ways of educating and developing health professionals (and allied staff such as healthcare assistants) so that they recognise, value and effectively support the efforts of the self-managing patient. At the very least, clinicians need to explore the details of their patients' home, work/school and community environments more, so that consideration of these can be factored into any 'goal setting' and management plans. We also need to work on removing barriers to effective diabetes self-management in wider society. Voluntary agreements with the food industry might be less effective than legislative changes, but in the absence of the latter, higher standards for nutritional information in cafés and restaurants should be strongly encouraged.

### Suggestions for further research

Whilst we have claimed some theoretical generalisability (we have shown, for example, that there is a recursive, mutually reinforcing, relationship between wider social determinants of health and individual action in relation to self-management), we do not claim that the findings from this small study are empirically generalisable. A number of themes from our findings should be explored further in empirical studies. A larger sample of participants, perhaps broken down by ethnic and demographic subgroups, studied more intensively for a longer period, would almost certainly reveal important additional insights into the challenges of self-management, though the acceptability and resource implications of an extended ethnographic approach may be prohibitive. The use of design tools such as 'cultural probes', which engage research participants in collecting ethnographic data in the absence of the researcher [54], could take self-management research into fruitful new directions. Such studies might inform the development and evaluation of multi-level intervention programmes which address the complex needs of individuals who find self-management challenging. Ethnographic studies of diabetes in the workplace might be undertaken in partnership with occupational health departments.

There is also a need for further theoretical and methodological work on what might be termed the political economy of self-management. Tudor Hart's inverse care law states that because of pervasive economic and socio-cultural forces, people most in need of health care are least likely to seek it or receive it [55]. The findings from this preliminary study suggest that because of the pervasive impact of economic and socio-cultural forces on the opportunities for health-related actions, people who would benefit most from self-management of their diabetes may be least likely to achieve it. Social ecology models of risk and chronic illness [47], perhaps combined with a 'critical theory' application of ethnographic methods [56], could help take this agenda forward. A bold and innovative approach to researching self-management is needed not least because without it, the social determinants of poor diabetes outcome are likely to exert their impact in the next generation as well as the present one.

### Funding, governance and ethical approval

The study was initially conceptualised (and data collection commenced) as a component of a study funded by the Department of Health under the Connecting for Health Evaluation Programme and reported previously [57]. We subsequently extended our sample and analysis using funding from a Senior Investigator Award for TG from the National Institute of Health Research. The work was overseen by an External Advisory Group chaired by a layperson with representation from key stakeholders including patients, professional bodies and external academics. Ethical approval was obtained from two Multi-centre Research Ethics Committees: Thames Valley in January 2007 (06/MRE12/81 and subsequent amendments) and North West 8 in September 2009 (09/H1013/36 and subsequent amendments). The sponsor had no role in the preparation or submission of the paper.

## Competing interests

All authors have completed the Unified Competing Interest form at http://www.icmje.org/coi_disclosure.pdf (available on request from the corresponding author) and declare: no support from any organisation for the submitted work [or describe if any]; no financial relationships with any organisations that might have an interest in the submitted work in the previous three years [or describe if any], no other relationships or activities that could appear to have influenced the submitted work.

## Authors' contributions

TG conceptualised the study. SH did the fieldwork. Both authors analysed the data. TG led on writing the paper with input from SH. Both authors read and approved the final manuscript.

## Pre-publication history

The pre-publication history for this paper can be accessed here:

http://www.biomedcentral.com/1472-6963/12/83/prepub
